# Impact of Coronary Tortuosity on Coronary Pressure: Numerical Simulation Study

**DOI:** 10.1371/journal.pone.0042558

**Published:** 2012-08-14

**Authors:** Yang Li, Zhengtao Shi, Yan Cai, Yi Feng, Genshan Ma, Chengxing Shen, Zhiyong Li, Naifeng Liu

**Affiliations:** 1 Department of Cardiology, Zhongda Hospital, School of Medicine, Southeast University, Nanjing, China; 2 School of Biological Science and Medical Engineering, Southeast University, Nanjing, China; 3 Department of Cardiology, Xinhua Hospital, Shanghai Jiao Tong University, Shanghai, China; Indiana University School of Medicine, United States of America

## Abstract

**Background:**

Coronary tortuosity (CT) is a common coronary angiographic finding. Whether CT leads to an apparent reduction in coronary pressure distal to the tortuous segment of the coronary artery is still unknown. The purpose of this study is to determine the impact of CT on coronary pressure distribution by numerical simulation.

**Methods:**

21 idealized models were created to investigate the influence of coronary tortuosity angle (CTA) and coronary tortuosity number (CTN) on coronary pressure distribution. A 2D incompressible Newtonian flow was assumed and the computational simulation was performed using finite volume method. CTA of 30°, 60°, 90°, 120° and CTN of 0, 1, 2, 3, 4, 5 were discussed under both steady and pulsatile conditions, and the changes of outlet pressure and inlet velocity during the cardiac cycle were considered.

**Results:**

Coronary pressure distribution was affected both by CTA and CTN. We found that the pressure drop between the start and the end of the CT segment decreased with CTA, and the length of the CT segment also declined with CTA. An increase in CTN resulted in an increase in the pressure drop.

**Conclusions:**

Compared to no-CT, CT can results in more decrease of coronary blood pressure in dependence on the severity of tortuosity and severe CT may cause myocardial ischemia.

## Introduction

Coronary tortuosity (CT) is a common coronary an5giographic finding, it has been reported that CT may be associated with angina pectoris [1], [2] and eversible myocardial perfusion defects [3], weather CT can lead to cardiac ischemia by diminishing coronary pressure has not yet been clarified. The present study was designed to determine the impact of CT on coronary pressure by numerical simulation.

## Methods

**Figure 1:**
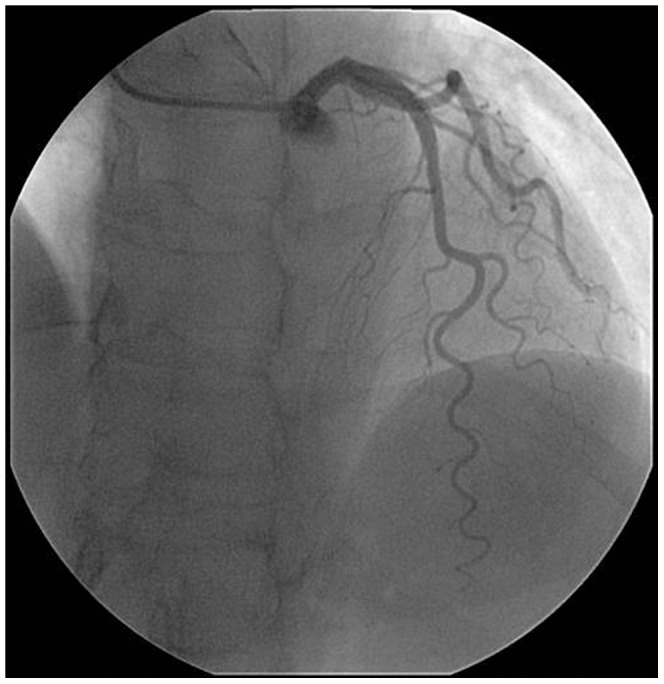
Angiography of LAD tortuosity.

Figure 1 was an example of coronary artery angiography showing CT of the left anterior descending artery (LAD). Two morphological parameters were considered to represent the tortuosity: coronary tortuosity angle (CTA) and coronary tortuosity number (CTN). To investigate the effects of CTA and CTN on the pressure distribution along the tortuous artery, 2D idealized models with the CTA of 30°, 60°, 90°, 120° and CTN of 0, 1, 2, 3, 4, 5 were established using software Gambit 2.2.30.

### Geometry of the models and mesh

**Figure 2:**
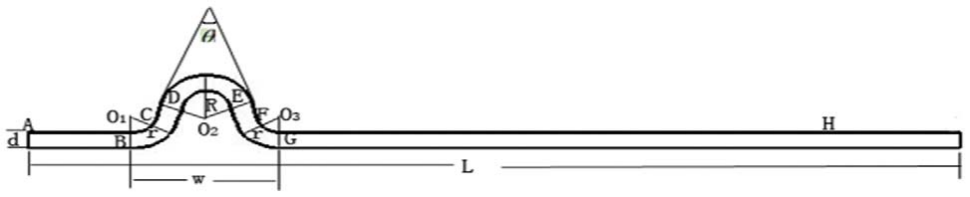
Geometrical parameters of model (CTA = 30°, CTN = 1).

Adopted from the in vivo coronary artery angiography (Fig. 1), the model for CTA = 30°, CTN = 1 was shown in Figure 2. The total length of coronary artery (denoted by L) was 130 mm and the diameter (denoted by d) was 3 mm. The width of one tortuous artery (denoted by w) was 20 mm. R and r were assumed to be 6.318 mm, 6 mm, respectively. *θ* was the CTA. We changed the angle and the number of tortuous coronary artery to obtain other models.

The mesh element was quadrilateral and a pave mesh was applied on the whole artery region. The interval size was 1 mm. The number of the quadrilateral cells was 429.

### Model parameters and boundary conditions

Blood was assumed to be incompressible, homogeneous and Newtonian fluid, which was a valid assumption in large arteries if the diameter of the artery exceeded 6 mm. The blood viscosity was chosen as 0.0035 Pa.s and the density was 1050 kg/m^3^ [4], [5].

The boundary conditions included: (a) no slip condition on the wall [6], (b) the wall was rigid without displacement, (c) the inlet velocity was 0.156 m/s (LAD baseline average peak velocity) [7] and the outlet pressure was zero [8], [9] for steady condition (d) the inlet velocity and the outlet pressure were both time-dependent waveform for the pulsatile simulation.

### Computational fluid dynamics

The blood flow satisfied the Navier-Stokes equations. Finite Volume CFD code Fluent 6.2.16 Inc was used to solve the equations to get the solution of the distribution of pressure and velocity. SIMPLE was chosen to solve the couple of velocity and pressure, the pressure equation was Standard and the momentum equation was First Order Upwind. The convergence criterion was set to be 10^−3^.

## Results

### Hemodynamic parameters under steady condition

**Figure 3:**
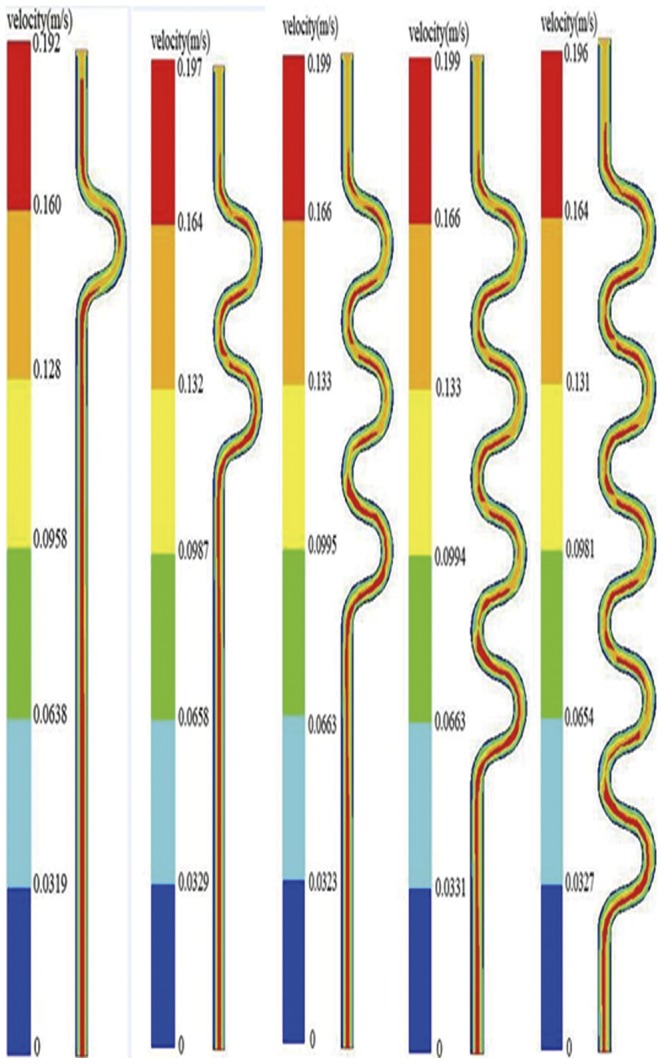
The distribution of velocity with different CTN (CTA = 30°).

Blood velocity was closely associated with the pressure distribution. Figure 3 showed the velocity changes with different CTN when CTA was 30°. The Reynolds number in current models was 154, which was much smaller than the low limitation of turbulent flow (Reynolds number = 2300). Thus the blood flow was laminar and the maximum velocity located near the centerline of the vessel, and the velocity along the artery wall was nearly zero.

**Figure 4:**
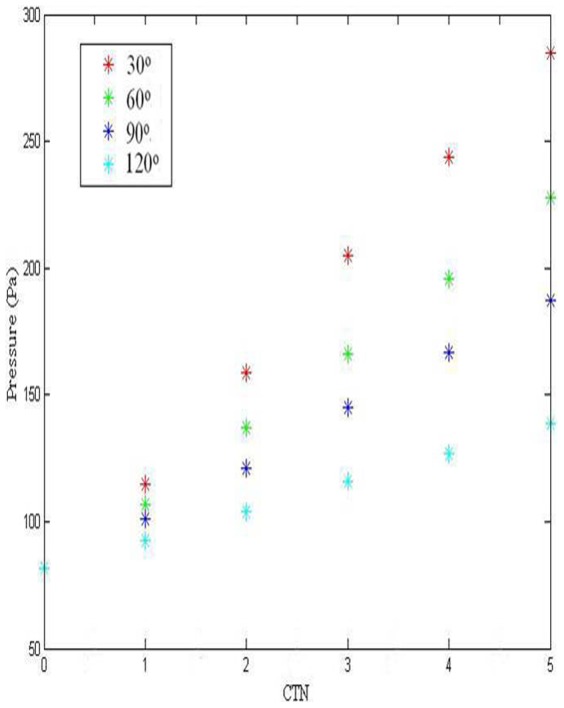
Pressure drop at different CTN and CTA.

We calculated the pressure drop between the start and the end of tortuous artery in different models, as shown in Table 1 and Figure 4. The pressure drop was positively associated with CTN but negatively associated with CTA, which indicated that more severity of CT may induce more pressure drop along the coronary artery. The variation trends at different CTN and CTA were presented in Figure 4. It can be seen that the pressure drop increase linearly with CTN, and this association is more profound for a smaller value of CTA.

**Table 1:** Pressure drop (Pa) at different CTN and CTA under steady condition.

	CTN = 0	CTN = 1	CTN = 2	CTN = 3	CTN = 4	CTN = 5
CTA = 30°	81.5	115	159	205	244	285
CTA = 60°	81.5	107	137	166	196	228
CTA = 90°	81.5	101	121	145	167	187
CTA = 120°	81.5	92.7	104	116	127	139

**Figure 5:**
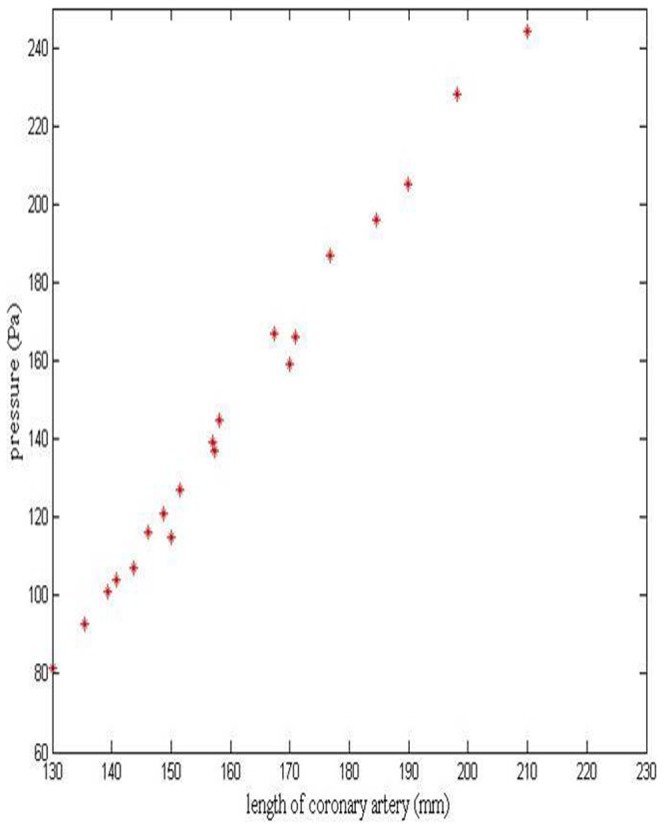
Pressure drop at different length of coronary artery.

Figure 5 shows the relationship between the length of coronary artery and pressure drop. The length of coronary artery is associated with both CTA and CTN, which can be used as an essential index to reflect the severity of CT. The pressure drop increases almost linearly with the length of artery.

### Analysis of pressure distribution under pulsatile simulation

**Figure 6:**
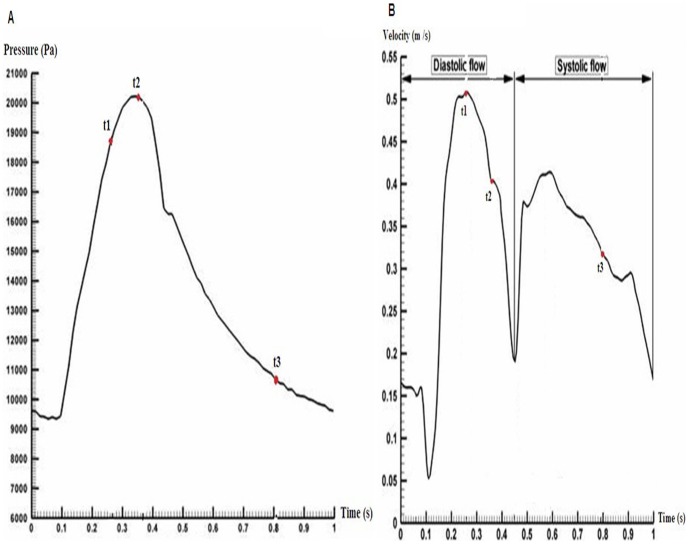
Boundary conditions under pulsatile state. (A) Inlet velocity curve in one cardiac cycle, (B) Outlet pressure curve in one cardiac cycle.

Figure 6 was the representative time-dependent inlet velocity and outlet pressure [10], which was used in our pulsatile simulation. The time point t1, t2 and t3 are three important stages in the cardiac cycle, which corresponds to the peak diastolic, the maximum pressure and the late systolic. Numerical simulation was performed for three cardiac cycles and the results at the second cycle were chosen to analyze the pressure drop for different models.

**Figure 7:**
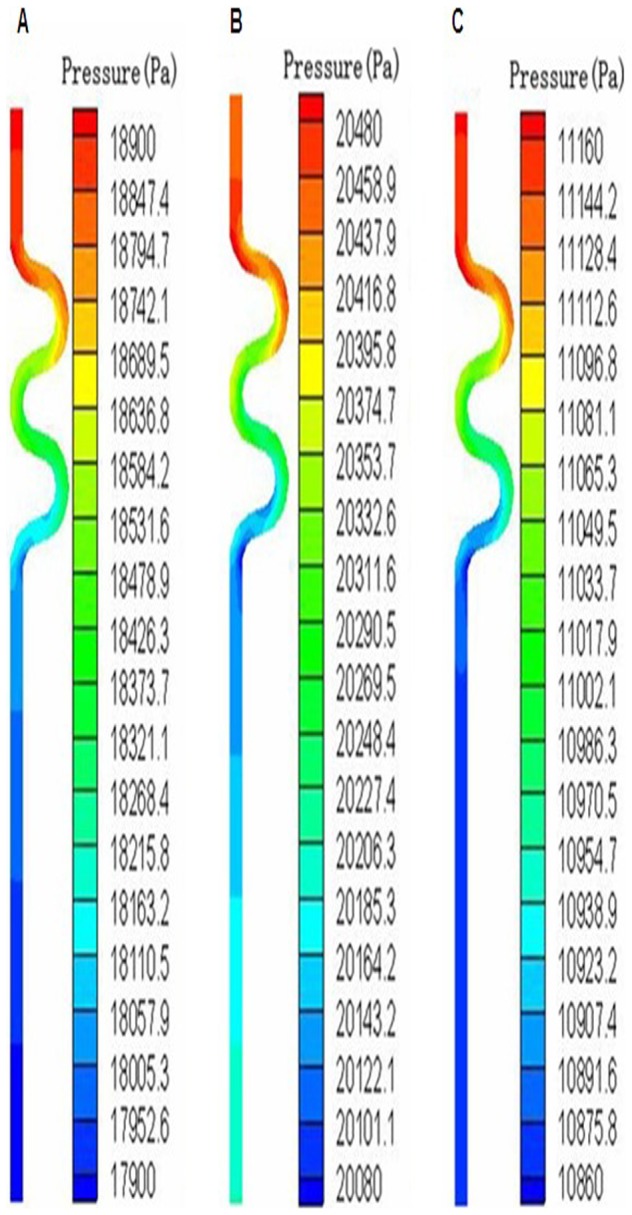
Pressure distribution at three time points in one cardiac cycle (CTA = 30o and CTN = 2). (A) Blood pressure variation at t1, (B) Blood pressure variation at t2, (C) Blood pressure variation at t3.

Figure 7 was the pressure change at the three time points when CTA was 30° and CTN was 2. The inlet velocity and the outlet pressure were 0.51 m/s and 17850 pa, 0.42 m/s and 20206 pa, 0.318 m/s and 10869 pa for t1, t2 and t3 respectively. Pressure distribution was very similar to the results under steady condition, and the pressure drop was 1000 pa, 400 pa and 300 pa respectively. Table 2 shows the pressure changes for the models with different CTN and CTA at the three time points. The pressure drop increased with CTN but decreased with CTA, which was also similar to the results under the static condition.

**Table 2:** Pressure drop (Pa) with different CTN and CTA at three time points under pulsatile condition (t1 = 0.26 s, t2 = 0.36 s, t3 = 0.8 s).

	t	CTN = 1	CTN = 2	CTN = 3	CTN = 4	CTN = 5
CTA = 30°	t1	550	1000	1400	1700	2000
	t2	200	400	600	700	900
	t3	150	300	400	550	750
CTA = 60°	t1	450	750	1000	1300	1500
	t2	200	300	400	550	600
	t3	130	220	320	400	450
CTA = 90°	t1	460	600	800	1000	1200
	t2	150	200	320	400	500
	t3	100	180	260	340	380
CTA = 120°	t1	360	400	500	600	1100
	t2	140	140	170	180	220
	t3	65	110	140	180	200

## Discussion

The present investigation demonstrated that CT can result in more decreases of coronary pressure compared to no-CT. CT is a common coronary angiographic finding in patients with angina. It has been reported that CT may be positively related to age, cardiac shrinkage [11], hypertension [12], [13], impaired left ventricular relaxation [14], and negatively correlated with coronary atherosclerosis [12], [15] and cardiac enlargement [11], but the relationship between CT and coronary ischemia is still unclear.

The current study is the first to investigate the impact of CT on coronary pressure quantitatively. Little attention has been paid on the impact of arterial tortuosity on blood pressure before. It was found that tortuosity of internal carotid artery results in a decrease of blood pressure in the distal segment of tortuous internal carotid artery in dependence on the angle of tortuosity, and the decrease is obvious when the angle of tortuous artery is less than 30° [16]. Our study also shows that CT can lead to a decrease of coronary pressure in dependence on the severity of tortuosity, and severe CT can cause apparent reduction in coronary pressure (15 mmHg) and may cause myocardial ischemia. Ventricular hypertrophy might affect the geodesic pattern of coronary arteries, and an increase in coronary flow might stimulate growth in coronary caliber, length, and collateral growth when left ventricle is hypertrophic [17]. Coronary circulatory regulation is well-known as auto regulation in response to hypoxia. Blood pressure of tortuous coronary artery can be measured by intracoronary pressure wire [18], [19], and coronary flow reserve of tortuous coronary artery should be assessed by transthoracic doppler echocardiography [20] or intracoronary Doppler [7].

We have to clarify that this study has some limitations because a few assumptions were made to simplify the simulation. First, the heart movement and the movement of the coronary arteries due to the myocardial tension cannot be simulated yet, therefore, not included in this study. Second, we also neglected the influence of tissues and organs surrounding the coronary artery during the modeling and simulation [10]. Third, the wall was thought to be rigid and no slip which indicated zero radial, axial velocity at the wall and no extension of the wall length [20], while coronary artery wall deformation in the axial and circumferential directions were always changing in human body.

The following directions may be further investigated in the future. First, as the change of the blood pressure in the cardiac cycle could cause significant displacement of the coronary artery wall, the interactions between the blood flow domain and blood vessel wall were strongly coupled [5]. Intraluminal thrombus (ILT) and calcification which can affect the hemodynamic parameters clearly [21], [22] often exist in the artery wall, so choosing suitable linear elastic or hyperelastic material models for ILT, calcification and artery wall will provide accurate results by the fluid-structure interaction (FSI) model. Second, 3D patient-specific models based on imaging of the coronary artery would be more realistic. Third, in the vitro experiments or animal models will be very useful to validate our computational simulation.

In conclusion, CT can result in more decrease of coronary blood pressure in dependence on the severity of tortuosity, severe CT may cause myocardial ischemia. Further studies are needed to investigate the coronary flow reserve of tortuous coronary artery.
